# Elephant Movement Data Provides Insights Into Conservation Challenges and Successes in the Ruaha–Rungwa Landscape

**DOI:** 10.1002/ece3.72197

**Published:** 2025-09-30

**Authors:** Ramadhan Juma, Alfred Kikoti, Kristen D. Snyder, Eivin Røskaft, Peter S. Ranke, Han Olff, Alex Lobora, Godwell Ole Meing'ataki, Michael H. Kimaro

**Affiliations:** ^1^ Tanzania World Elephant Centre Arusha Tanzania; ^2^ Department of Fish, Wildlife, and Conservation Biology Colorado State University Fort Collins Colorado USA; ^3^ Grumeti Fund Mugumu Mara Region Tanzania; ^4^ Department of Biology Norwegian University of Science and Technology Trondheim Norway; ^5^ Gjaerevoll Centre for Biodiversity Foresight Analyses Norwegian University of Science and Technology Trondheim Norway; ^6^ BirdLife Norway Trondheim Norway; ^7^ Groningen Institute for Evolutionary Life Sciences University of Groningen Groningen the Netherlands; ^8^ Tanzania Wildlife Research Institute Arusha Tanzania; ^9^ Tanzania National Parks Arusha Tanzania; ^10^ Tanzania Research and Conservation Organization Morogoro Tanzania

**Keywords:** elephant speed, elephants, GPS‐satellite technology, protection levels, Ruaha–Rungwa ecosystem

## Abstract

The African savannah elephant ( *Loxodonta africana* ) is listed as endangered on the IUCN Red List. The Ruaha–Rungwa ecosystem supports Tanzania's largest elephant population and remains a critical yet understudied stronghold for elephant conservation in East Africa. Effective conservation in this ecosystem requires understanding elephant movements across large landscapes and varying levels of disturbance. This study addressed the gap by utilizing GPS‐satellite tracking of 28 elephants over a 4‐year period in Tanzania's Ruaha–Rungwa ecosystem. It examined their home ranges and movement patterns inside and outside protected areas and identified key elephant hotspots. The findings aim to improve conservation strategies and mitigate human–elephant conflicts through better habitat management and protection efforts. The study found no significant difference between home range estimates from Minimum Convex Polygon and Adaptive Kernel Density Estimation, with 28 collared elephants averaging 2536 km^2^ at the 99% isopleth. Home range size varied by age and season but not by sex. Elephants moved faster at night in less protected areas during both wet and dry seasons. Population‐level habitat use was similar across protection levels. Village lands in western Rungwa Game Reserve and its surrounding areas, as well as the eastern regions outside Ruaha National Park, served as key refuge sites during the wet season. Findings suggest elephants in the Ruaha–Rungwa ecosystem require vast areas, with adjacent lands playing a crucial role in their survival. High elephant activity in village lands within the western Rungwa Game Reserve and Lunda‐Mkwambi Game Controlled Area highlights the potential for human–elephant conflict, emphasizing the need to reopen the wildlife corridor for linking Ruaha–Rungwa and Rukwa–Katavi ecosystems. This study offered crucial insights into elephant movement and behavior in a landscape increasingly shaped by human–elephant conflict and habitat fragmentation, informing strategies for connectivity, land‐use planning, and coexistence.

## Graphical abstract

The African savannah elephant, listed as vulnerable on the IUCN Red List, requires vast areas for survival, with adjacent lands playing a crucial role in its movement. This study used GPS tracking of 28 elephants in Tanzania's Ruaha–Rungwa ecosystem over 4 years, revealing that home range size varied by age and season but not by sex, and that elephants moved faster at night in less protected areas. Findings highlight key refuge sites in village lands and emphasize the need to reopen wildlife corridors to mitigate human–elephant conflicts and improve conservation strategies.

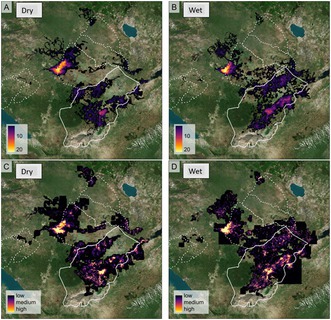

## 1 Introduction

Over the last 100 years, African savanna elephant ( *Loxodonta africana* ) populations have declined due to hunting for ivory and habitat loss (Wittemyer et al. 2014; Blanc and Barnes 2007). Despite this, African elephants still cover an extensive geographical range (Blanc and Barnes 2007), and some populations have seen a resurgence in numbers (Thouless et al. 2016). Continued conservation of elephant populations relies on understanding their behaviors, including how the movement and ranging behavior of elephants are influenced by various factors, including surface water (De Beer and van Aarde 2008; Ogutu et al. 2014), human impacts (Selier et al. 2015; Benitez et al. 2022), and vegetation productivity (Young et al. 2009). Home range is a key proxy for understanding elephant movement, space use, and foraging needs (De Beer and van Aarde 2008; Schoener 1981), and is influenced by factors such as age, sex, and environmental conditions. Adult female elephants, which tend to form stable social groups, are often used as representatives for studying movement patterns. However, males are more solitary or move in bachelor herds and small multi‐age groups (Owen‐Smith 1988; Hollister‐Smith et al. 2007). Studies have shown conflicting results on sex‐based differences in home range size, with some suggesting that males' ranges are larger (Ngene et al. 2017), while others report no difference (Wall et al. 2013). GPS tracking at high spatiotemporal resolution allows researchers to assess variability in movement speed and displacement, which are shaped by factors such as habitat type, forage availability, human disturbance, predation risk, and seasonality (Loarie et al. 2009; Graham et al. 2009; Boettiger et al. 2011). Movement speed influences foraging efficiency, predation risk, and energy use (Rowcliffe et al. 2016), while activity patterns reveal evolutionary adaptations and behavioral responses to human pressures (Zhang et al. 2017). Increasing nocturnality due to daytime human activities may help elephants avoid conflict, but it could disrupt their natural behaviors (Gaynor et al. 2018). Tracking data also helps identify elephant use hotspots, improve conservation planning, guide corridor establishment, and support upgrades to low‐protection areas (Hahn et al. 2022, 2023).

The Ruaha–Rungwa ecosystem is home to the largest elephant population in Tanzania and is one of the strongholds of elephants in East Africa (Jones et al. 2018). While elephant populations in northern Tanzania, such as those in the Serengeti (Snyder et al. 2021; Snyder et al. 2023) and Tarangire ecosystems (Galanti et al. 2006; Kioko et al. 2013), have received extensive research and conservation attention, central populations like those in Ruaha–Rungwa remain understudied despite facing increasing threats (Beale et al. 2018; Mkuburo et al. 2020; Smit et al. 2023). The ecosystem is characterized by a mix of protected areas and human‐dominated landscapes, making it a hotspot for human–elephant conflict, especially during the wet season when elephants move into village lands in search of food and water (Stommel et al. 2016; Hariohay et al. 2020). Understanding elephant movement, space use, and behavioral adaptations in this region is essential for developing targeted conservation strategies, mitigating conflict, and maintaining ecological connectivity (Larson et al. 2004; Seoane et al. 2006; Beier et al. 2011; Penjor et al. 2021), particularly through the threatened corridor linking Ruaha–Rungwa to the Rukwa–Katavi and Ruaha–Ugalla ecosystems (Hariohay et al. 2017, 2019, 2020; Lyakurwa et al. 2020). Given the vulnerable status of African savannah elephants and the growing human footprint in Tanzania, this study provides crucial insights to inform land‐use planning, enhance protection efforts, and promote coexistence between people and elephants in one of East Africa's most important conservation landscapes.

In this study, we analyzed tracking data from 28 collared elephants to assess their home ranges, movement behavior, and habitat utilization. Specifically, the objectives of this study were to (i) determine the home range characteristics of the collared animals and assess variability by sex and age class; (ii) evaluate spatial and temporal variation in elephant movement speed with different levels of protection status and time of day, and (iii) identify critical elephant habitat in the Ruaha–Rungwa ecosystem and discuss the implications for elephant conservation.

## 2 Materials and Methods

### 2.1 Study Area

**FIGURE 1:**
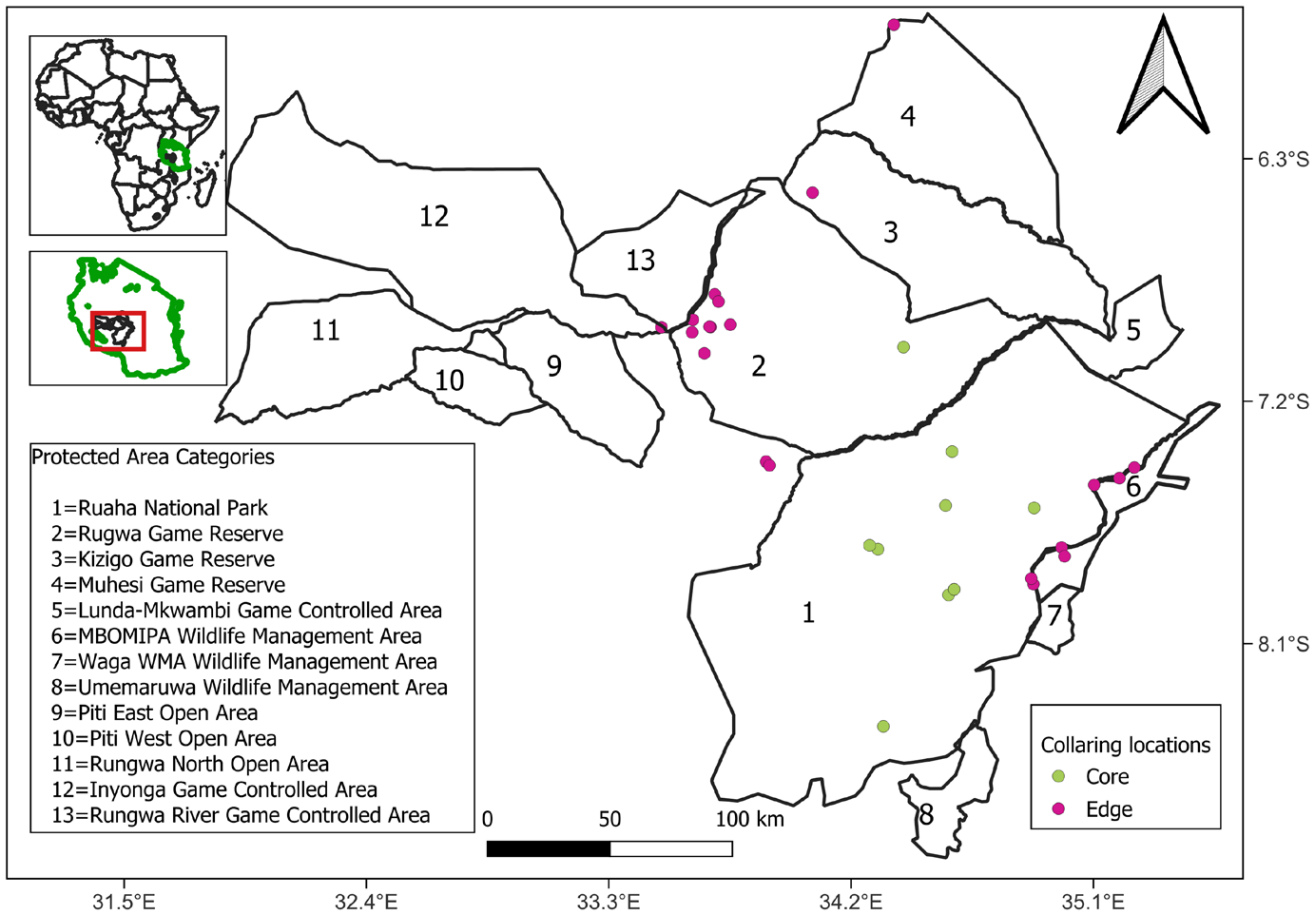
Map showing the location of 30 collared elephants that occurred in November 2015 in the core Ruaha National Park and Rungwa Game Reserve (indicated with “green dots”) and those collared at the edge (within 10 km from the boundary) of protected area boundaries (indicated with “red dots”).

The Ruaha–Rungwa ecosystem is situated in Southern‐Central Tanzania and comprises Ruaha National Park (RNP), Rungwa, Kisigo, and Muhesi Game Reserves (RKM GRs), Lunda‐Mkwambi Game Controlled Area, MBOMIPA, and Umemarua Wildlife Management Areas, and surrounding unprotected human settlement area (Figure 1). In combination, these protected areas cover approximately 50,000 km^2^ (Jones et al. 2018). Ruaha National Park is the second‐largest national park in Tanzania. It is one of the most significant ecoregions in the world, given its abundance and diversity of plants and animals (Olson and Dinerstein 1998). The RKM GRs are primarily located in Manyoni, within the Singida Region (98%) in central Tanzania (Mkuburo et al. 2020). These three reserves are managed as a single entity, with headquarters located in the Rungwa village. The Ruaha–Rungwa ecosystem has vegetation typical of the East African savanna and miombo woodland (Abade et al. 2014; Cusack et al. 2015). The area is home to a substantial population of African lions ( *Panthera leo* ), African elephants, and lesser kudu ( *Tragelaphus imberbis* ), among other wildlife species (Cusack et al. 2015).

The climate of the area is semi‐arid to arid, characterized by dry seasons that typically occur from May to November, and wet seasons from December to April. The mean annual rainfall is 873 mm (Hariohay et al. 2019) and the temperature ranges from 15°C to 35°C (Abade et al. 2014). The main commercial activity in the park is non‐consumptive tourism. At the same time, the adjacent protected areas, such as game reserves, game‐controlled areas, and wildlife management areas, allow both photographic tourism and trophy hunting (United Republic of Tanzania (URT) 2009). The human population growth around the Ruaha–Rungwa ecosystem increases annually at a rate between 2.4% and 3.9% (The United Republic of Tanzania (URT) 2022). Wildlife crimes such as bushmeat, timber logging, elephant poaching, livestock grazing, and illegal mining occur at high levels, especially around Rungwa Game Reserves (Hariohay et al. 2017). Local communities living adjacent to the protected areas are mainly engaged in crop cultivation, livestock keeping, beekeeping (Dickman 2009; Hariohay et al. 2017; Kimaro and Hughes 2023), and fishing around the Lunda‐Mkwambi Game Controlled Area near Mtera dam (Bayo and Rija 2021).

### 2.2 Data Collection

In November 2015, a total of 30 elephants were fitted with GPS collars (Vectronic GPS‐Iridium, with an accuracy of 8–10 m) within the Ruaha–Rungwa ecosystem. Thirteen collars were deployed to individuals found inside Ruaha National Park, and 17 collars were deployed near the edge (within 10 km from the boundary) of the park, adjacent game reserves, and WMAs. Collars were distributed across bulls and cows but were biased toward the latter (*n* = 21) because a primary goal of this deployment was to understand the movement of elephants, and a single‐collared cow is representative of the movements of entire cow‐calf groups (Loarie et al. 2009). Collars were preferentially deployed on tuskless individuals (*n* = 20) to reduce the risk of losing collars to poachers (Wasser et al. 2015; Thouless et al. 2016). All 30 collars were deployed over 11 days from 4th to 15th November 2015 (Supporting Information S1). The collared elephants were immobilized using etorphine (M99: C‐vet UK) and administered using a dart gun from a helicopter. Once the elephant was recumbent, it was fitted with a Vertex telemetry unit, and physical and health status measurements were taken. The immobilized elephants were then revived using diprenorphine (M5050: C‐vetUK). All elephants survived the collaring procedure with no injuries or deaths.

Thirty GPS collars were deployed from November 2015 to September 2019. Animals were collared for a maximum of 46 months. For this study, we included collars with at least 1 year of data (*n* = 28). The age distribution of collared elephants was as follows: two individuals were aged 25, nine individuals were aged 30, nine individuals were aged 35, and eight individuals were aged 40 years. Collars were programmed to attempt GPS fixes at every hourly interval. A total of 191,703 fixes were recorded during the study period. The fixed success rate was 21% (Table A1). The low fix rate from the GPS collars was due to technical errors.

### 2.3 Data Analysis

#### 2.3.1 Home Range Estimation

Although the use of probabilistic methods is highly recommended in the estimation of the animal home range (Börger et al. 2006), researchers still acknowledge conventional deterministic methods, especially the Minimum Convex Polygon (MCP) in home range estimation (De Boer et al. 2005; Ngene et al. 2017). In this study, we used both deterministic (MCP) and probabilistic estimators (Adaptive Kernel Density Estimates [KDE], with normal or reference bandwidth as a smoothing parameter) to determine the overall home range of all 28 individuals at 50%, 95%, and 99% isopleths. To assess the effect of estimator type, two linear mixed‐effect models with scaled home ranges were developed. We used KDE to estimate home ranges, as it accounts for irregular time intervals associated with the low GPS fix success rate observed in this study. Collar ID was treated as a random effect variable. Fixed variables included in the model were season, sex, and age of individuals. Age was not formally classified; instead, estimated ages from the individuals observed during the collaring process were used, with individuals > 30 years considered older and those < 30 years considered younger (Hollister‐Smith et al. 2007). Mean NDVI values across the elephant range during the study period were used to define wet and dry seasons. The resulting models were compared by using the ANOVA function to determine the most parsimonious model.

#### 2.3.2 Movement Speed

Movement speed was calculated in kilometers per hour using the adehabitatLT package in R (Calenge 2006). Fixes that were likely to be errors (speed > 9 km/h) or with a lag greater than 4 h were excluded. We compared movement speed by protection level, season, and time of day. The protection level was classified as high (National Park), medium (Game Reserve), or low (Wildlife Management Area, Game Controlled Area, Open Area, Village Land) based on the location at the start of a given step. The time of day was defined broadly as either night (7 p.m.–7 a.m.) or day (7 a.m.–7 p.m.). We examined movement speed across these variables in isolation and combination using the linear mixed models (LMM) to determine whether movement speed significantly differed across protection level, season, and time of day.

#### 2.3.3 Habitat Utilization

To examine elephant space use, we constructed dynamic Brownian bridge movement models (dBBMMs). The dBBMM is an approach to model an individual's home range and utilization density that explicitly accounts for the temporal structure in the data and location error (Horne et al. 2007). This approach is particularly suitable for movement tracks with unequal sampling rates due to missing fixes, such as is the case with our dataset.

We constructed dBBMMs for each of the two seasons (dry and wet) of the year. We only included individual season years with at least 60 days of data in a season year (wet: *n* = 93, dry: *n* = 88). We excluded timesteps where the lag between fixes exceeded 8 h due to the high level of uncertainty regarding the animal's location and its impact on the required processing extent and time. Fixes where the speed exceeded 9 km/h were assumed to be location errors and were excluded (Bastille‐Rousseau et al., 2019). Models were constructed with a window size of 13 h and a margin of 3, using values relevant to the anticipated time scale of elephant behavioral shifts (Kranstauber et al., 2012).

We examined two measures of intensity of use at the population level. First, we determined the total count of individual seasons utilizing a pixel in the wet or dry season. We derived a presence‐absence layer for each season year from the 99% isopleth home range and summed the number of individual season years where presence was actual for each pixel. Given the low fixation rates observed in this study, we used a 99% isopleth to capture nearly all locations, including occasional long‐distance or exploratory movements. Next, we produced seasonal population‐level distribution maps by summing the utilization distributions for each season and normalizing the results. All statistical analyses were performed using R version 4.2.2 (R Core Team 2024).

## 3 Results

### 3.1 Home Range

**FIGURE 2:**
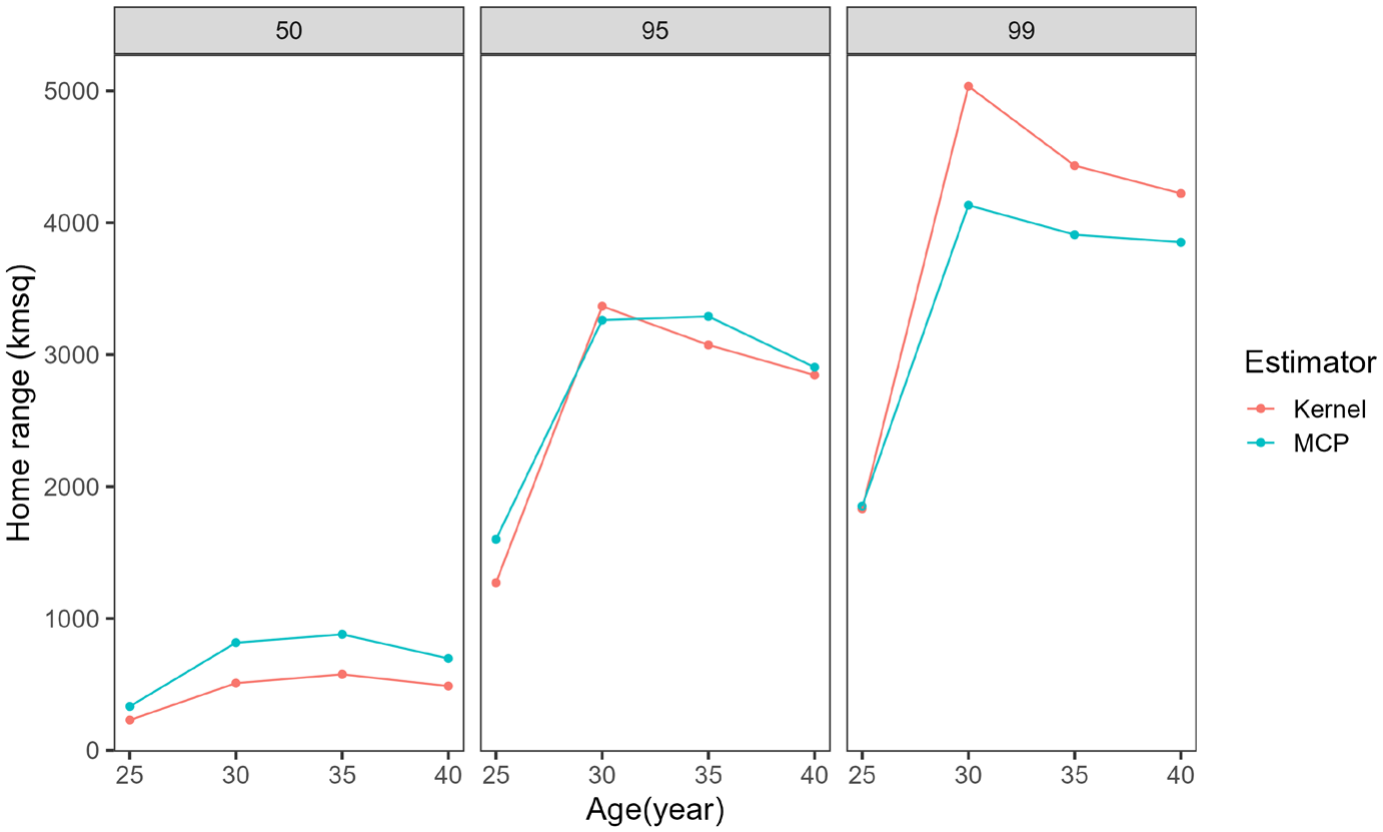
Home range estimation between dry and wet seasons, according to the age structure of individual elephants categorized based on 50%, 95%, and 99% isopleths of all movement points.

There was no statistical difference between MCP and Kernel density estimators (GLMM, *Z* = 1.135, *p* = 0.257). The overall average home ranges for all 28 collared elephants, as determined by combined MCP and Kernel density estimators at 50%, 95%, and 99% isopleths, were 624 ± 76 SE, 2932 ± 298 SE, and 3987 ± 384 SE km^2^, respectively (Table A1). The minimum and maximum home ranges for individual elephants at a 99% isopleth were 1818 and 25,539 km^2^. The overall home range for all individual elephants showed no significant difference between male and female individuals (GLMM, *z* = 0.294, *p* = 0.769); instead, it varied significantly between age structure and seasons (GLMM, *p* < 0.05, Table 1). Older elephants (> 30 years old) had a larger home range than young ones (< 30 years old), and the overall home range for all individual elephants was higher during the wet season than during the dry season (GLMM, *z* = 3.441, *p* = 0.001, Figure 2).

**TABLE 1:** Coefficients derived from the scaled linear mixed model showing the variation of home range with season, sex, and age structure of the collared elephants.

Predictors	Estimate	Standard error	*Z* value	*p*
Intercept	4.954	0.699	7.087	< 0.001
Male	0.086	0.299	0.288	0.774
Age 25	1.411	0.824	1.711	0.087
Age 30	1.869	0.712	2.624	0.009
Age 35	1.876	0.702	2.671	0.008
Age 40	1.882	0.676	2.784	0.005
Wet season	0.344	0.104	3.307	0.001

*Note:* The minimum age among all 28 collared elephants was 20 years old.

### 3.2 Movement Speed

**FIGURE 3:**
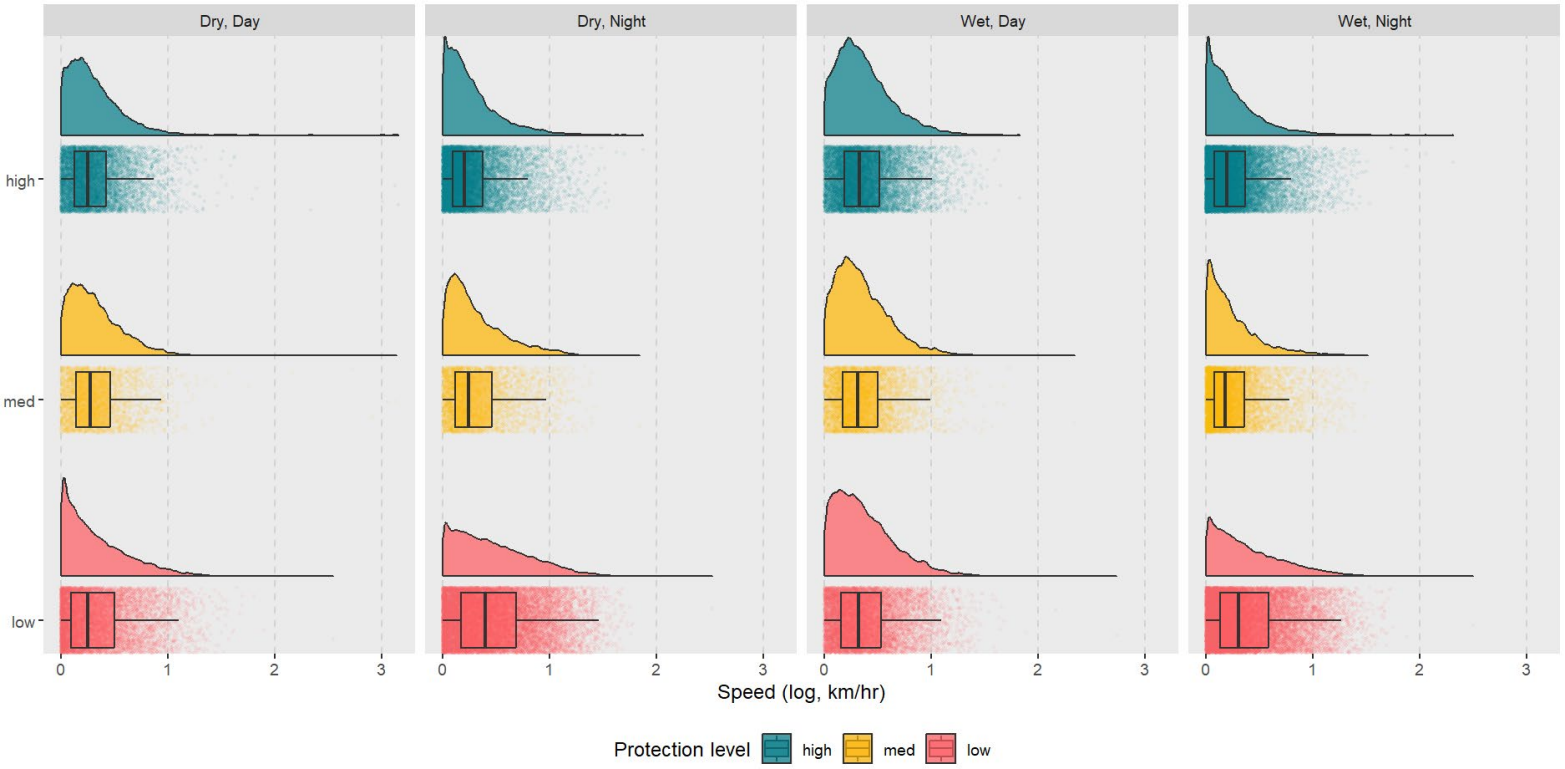
The distribution of elephant movement speeds was recorded via satellite collars by protection level, season, and time of day.

When examined in isolation, the average movement speeds were lower during the night than day (LMM, *t* = −8.06, *p* = 0.00). Speed was greater on average in low‐protection areas (LMM, *t* = 9.99, *p* = 0.00) than in medium and high‐protection areas. Speed was generally higher during the wet seasons than during the dry seasons (LMM, *t* = 15.91, *p* = 0.00). When accounting for season, nighttime speeds were lower on average than daytime speeds during the wet season, while the inverse was true during the dry season (LMM, *t* = −0.12, *p* = 0.00). When accounting for protection level, speeds were highest on average overnight in low‐protection areas (LMM, *t* = 33.19, *p* < 0.00) compared to medium‐ and high‐protection areas. Speeds were lowest overall overnight during the wet season in high and medium protection areas (LMM, *t* = −1.00, *p* = 0.32), while the speeds were highest overnight during the wet season in low protection areas (LMM, *t* = −11.22, *p* = 0.00; Table A2, Figure 3).

### 3.3 Habitat Utilization

**FIGURE 4:**
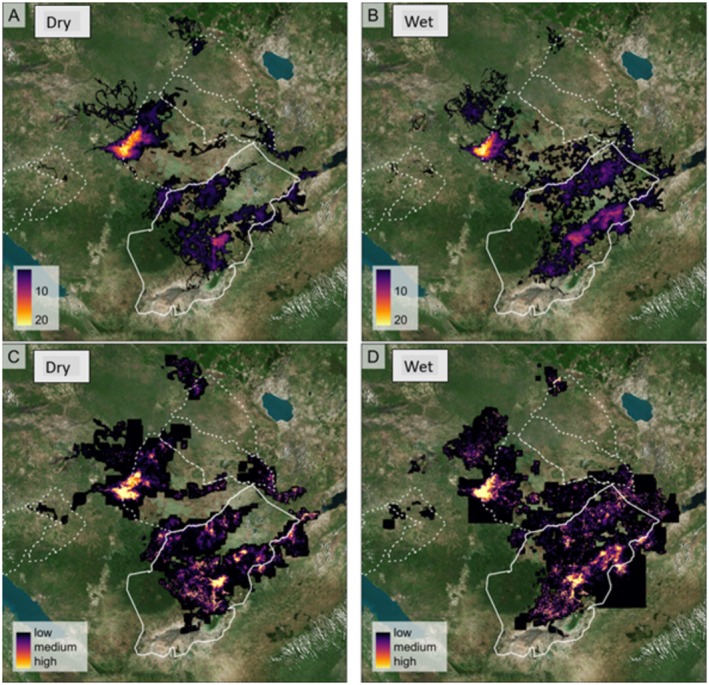
Elephant intensity of use in the Ruaha–Rungwa ecosystem as measured by the total count of elephant individual‐season‐years utilizing a given pixel in the dry (A) and wet (B) seasons, and overall population utilization in the dry (C) and wet (D) seasons. Boundaries of high‐protection areas (Ruaha NP) are shown with solid lines, and medium‐protection areas (game reserves) are shown with dashed lines.

The most significant and widely utilized areas for elephants were located in the core Ruaha National Park and the Rungwa Game Reserve and were generally similar for both dry (Figure 4A) and wet seasons (Figure 4B), based on total counts of individual elephant seasons within a given pixel. The overall utilization at the population level was primarily constrained to medium and high‐protection areas. Still, elephants utilize the low‐protected regions, especially the eastern and southern parts of the park, as well as the western Rungwa Game Reserve. Areas with low protection levels in the western Rungwa Game Reserve experienced high elephant utilization. The overall spatial extent utilized by elephants was observed to be greater during the wet season (Figure 4D) than during the dry season (Figure 4C), and this remained similar across protection levels.

## 4 Discussion

In this study, we observed that all collared elephants in the Ruaha–Rungwa ecosystem covered an overall average home range of 3987 km^2^, ranging from a minimum of 675 km^2^ to a maximum of 13,884 km^2^ at the 99% isopleth. These results indicate that elephants in this ecosystem moved in a large area and were relatively larger than the ranges reported from other savannah ecosystems like in Tarangire National Park (477–5060 km^2^; Galanti et al. 2006), Amboseli–Tsavo–Kilimanjaro ecosystem (6130–7025 km^2^; Ngere et al., 2016), Maputo reserve in Mozambique (169–453 km^2^; De Boer et al. 2005), Pongola reserve in South Africa (11–72 km^2^; Shannon et al. 2006), and Waza park in Cameroon (2484–3066 km^2^; Tchamba et al. 1994). In many landscapes, elephant home ranges are constrained by human activities (Huang et al. 2022); thus, the ability of elephants in this ecosystem to maintain extensive movements indicates relatively intact connectivity and functional habitat use. Although KDE accounts for irregular fix intervals (Van Kerm 2003), obtaining high‐resolution location data remains essential for improving the accuracy and detail of home range analyses in future studies.

Male and female elephants did not differ significantly in their home range size in the Ruaha–Rungwa ecosystem. These findings corroborate findings by Wall et al. (2013). However, our findings are contrary to those from several other areas, such as the Amboseli–Tsavo–Kilimanjaro ecosystem (Kikoti 2009; Ngene et al. 2017), and the Pangola reserve (Shannon et al. 2006), where they found a difference in home range with the sex of individual elephants. The home range of elephants varied with age, with older elephants, those more than 30 years old, exhibiting the larger home ranges. These findings align with De Villiers and Kok (1997), who demonstrated that breeding individuals tend to occupy larger areas than non‐breeding individuals. Many studies have found similar relationships between home range and seasons (Shannon et al. 2006; Young et al. 2009). In this study, the home range of elephants during the wet season was larger than during the dry season, indicating that elephant movements and habitat choices in the Ruaha–Rungwa ecosystem appear to be constrained by seasonal changes in surface water availability. Seasonal variation has been well documented previously (Ashiagbor and Danquah 2017; Shannon et al. 2006).

Overall, the movement speeds of elephants during the day were higher than at night. Some studies have reported that elephant movement speeds are higher during the day (Wall et al. 2013), while others have reported greater speeds at night (Loarie et al. 2009). The context of the level of protection and poaching risk is well studied, as our findings are consistent with the behavior of elephants moving more quickly when utilizing areas with high risk at night, especially in areas with low protection levels (Douglas‐Hamilton et al. 2005; Galanti et al. 2006; Harris et al. 2008; Gaynor et al. 2018), and when they are in cooler temperatures (Graham et al. 2009). The nighttime movement speed of elephants in this study was observed to be lower during the wet seasons in areas with all protection levels. Higher speed during the wet season was likely due to the presence of human disturbances during the wet seasons, such as bushmeat hunting, illegal timber logging, elephant poaching, illegal livestock grazing, and illegal mining (Hariohay et al. 2019), and likely due to difficulties in conducting anti‐poaching patrols during wet seasons (Nahonyo 2009).

We found that Ruaha National Park, Rungwa Game Reserve, and open areas/village lands found in western Rungwa Game Reserve were hotspots of elephant utilization in the Ruaha–Rungwa ecosystem. Historically, Ruaha National Park has been recognized as the most critical habitat for elephants within this ecosystem (Barnes and Douglas‐Hamilton 1982; Beale et al. 2018). Our results show that elephants utilize village lands bordering the western Rungwa Game Reserve. This corresponds with anecdotal reports that the villages bordering the western Rungwa Game Reserve are considered a corridor for migratory elephants moving between the Ruaha–Rungwa ecosystem and the Katavi–Rukwa ecosystem (Lobora et al. 2017). Historically, elephants have been identified as the most problematic animal in these villages (Hariohay et al. 2020), and illegal killing of wildlife, including elephants, is widespread in these villages (Beale et al. 2018; Hariohay et al. 2019). The open areas within and around these villages contain natural habitats that have the potential to maintain connectivity between the Ruaha–Rungwa and Rukwa–Katavi ecosystems (Lobora et al. 2017). We found that elephants frequently accessed these villages, but did not traverse from Ruaha–Rungwa to Rukwa–Katavi—this may indicate that the integrity of this corridor is highly threatened or no longer intact.

Land‐use planning is necessary to open and maintain the corridor (Lobora et al. 2017), as well as to develop economic and educational initiatives within the affected communities (Hariohay et al. 2019). Increased law enforcement could aid in protecting elephants when they try to pass through the corridor (Beale et al. 2018). Further work is needed to investigate elephant resource selection and the availability of forage, including its quality and quantity, to understand where elephant activity can be expected to occur throughout the year and in response to changing environmental conditions (De Knegt et al. 2011). These insights would facilitate the development of mitigation measures to reduce human–elephant conflicts in villages where the corridor of elephants enhances human–elephant coexistence.

## 5 Conclusions and Recommendations

In summary, based on tracking data from 28 elephants in the Ruaha–Rungwa ecosystem, elephants have an average home range of 3987 km^2^, which is larger than reported in other East African ecosystems. Elder elephants utilized larger rangelands than younger ones, and they moved more during the wet season, highlighting the underlying factors that intensify human–elephant conflict during this period; therefore, the development of appropriate mitigation methods is necessary. Elephants were at lower speeds inside the park and game reserves compared to areas with low protection levels, such as game‐controlled areas, wildlife management areas, open areas, and village lands. This suggests the need to enhance law enforcement efforts and reduce the impacts of increasing human–elephant interaction in regions with low protection levels. The core Ruaha National Park and Rungwa Game Reserve, along with village lands in western Rungwa Game Reserve, were key hotspot areas for elephants in the Ruaha–Rungwa ecosystem. This highlights the importance of establishing a wildlife corridor that connects the Ruaha–Rungwa ecosystem to the adjacent ecosystems.

We recommend continuing to protect Ruaha National Park and its adjacent protected areas. Measures to address human–elephant conflict in the villages are necessary, especially in the north‐western part of Rungwa Game Reserve and Lunda‐Mkwambi game‐controlled area, as it harbors potentially important habitats for elephants. Improving anti‐poaching efforts in both dry and wet seasons is essential in areas with low protection levels. Medium and high protection levels reveal that elephants exhibit increased speed during the night and dry seasons, which brings the need to confirm whether trophy hunting or illegal poaching has contributed to this. Investigating why elephants utilize village lands by examining their resource selection and the availability of forage, in terms of both quality and quantity, is a crucial step in mitigating human–elephant conflict and enhancing human–elephant coexistence.

## Author Contributions

**Ramadhan Juma:** conceptualization (lead), data curation (equal), funding acquisition (equal), investigation (equal), methodology (equal), project administration (equal), resources (equal), software (equal), supervision (equal), validation (equal), visualization (equal), writing – original draft (equal), writing – review and editing (equal). **Alfred Kikoti:** conceptualization (equal), data curation (equal), funding acquisition (equal), investigation (equal), methodology (equal), project administration (equal), resources (equal), software (equal), supervision (equal), validation (equal), visualization (equal). **Kristen D. Snyder:** formal analysis (equal), writing – review and editing (equal). **Eivin Røskaft:** data curation (equal), writing – original draft (equal), writing – review and editing (equal). **Peter S. Ranke:** formal analysis (equal), writing – original draft (equal), writing – review and editing (equal). **Han Olff:** writing – review and editing (supporting). **Alex Lobora:** validation (equal), visualization (equal), writing – review and editing (equal). **Godwell Ole Meing'ataki:** conceptualization (equal), funding acquisition (equal), methodology (equal), resources (equal), software (equal), supervision (equal), writing – review and editing (equal). **Michael H. Kimaro:** data curation (equal), formal analysis (equal), writing – original draft (equal), writing – review and editing (equal).

## Ethics Statement

It is hereby consciously declared that this material is the author's original work, which has not been previously published elsewhere. The paper accurately reflects the author's research and analysis. The paper properly credits the meaningful contributions of co‐authors and co‐researchers. All animal handling and collaring operations were conducted under the permit of relevant research management institutions and wildlife regulatory authorities. The animal ethics committee from the Tanzania Wildlife Research Institute (TAWIRI) approved the collaring of elephants. Qualified veterinarians from TAWIRI, in collaboration with veterinarians from Tanzania National Parks (TANAPA), conducted tranquilization, while the World Elephant Centre ecologist handled the elephants' collaring activity.

## Conflicts of Interest

The authors declare no conflicts of interest.

## Supporting information


**Data S1:** ece372197‐sup‐0001‐DataS1.docx.

## Data Availability

Data and R codes used in this manuscript have been shared and can be accessed as supporting material via the following link: https://drive.google.com/drive/u/0/folders/15iKolhTyoTT5SjxqkhMb69rkp5NoHwzb.

## Appendix A

**TABLE A1:** The home range of 28 collared elephants was calculated as an average at 50%, 95%, and 99% isopleths combined for both estimator types, with their age and sex.

ID	Age	Sex	Expected fixes	Received fixes	Fixes received (%)	50% home range	95% home range	99% home range
19880	25	♀	33,120	9568	29	760	4350	5493
19881	40	♂	33,120	4527	14	566	5937	9257
19882	35	♀	33,120	4931	15	2829	8760	11,198
19883	30	♀	33,120	7583	23	290	1510	2020
19884	35	♀	33,120	5723	17	1151	4886	6477
19885	40	♂	19,167	2663	14	1596	4783	6429
19886	40	♂	17,020	5885	18	440	2066	3073
19887	40	♀	33,120	2583	8	483	2449	3279
19888	35	♀	33,120	6933	21	481	1396	1818
19889	30	♀	33,120	12,294	37	241	1643	2172
19890	30	♀	33,120	1473	5	1511	11,724	15,118
19891	30	♀	33,120	17,935	54	3453	15,420	18,638
19892	30	♀	33,120	9120	28	1211	4411	5814
19893	30	♀	33,120	3883	12	1313	4630	5798
19894	35	♀	33,120	5635	17	316	3371	4513
19895	35	♀	33,120	10,735	32	536	2843	4073
19896	25	♀	33,120	6823	21	368	1390	1875
19897	30	♀	33,120	2636	8	1828	8474	10,783
19899	40	♀	33,120	4010	12	3464	14,833	18,882
19900	40	♂	19,167	7841	41	415	2784	4931
19901	35	♀	33,120	4912	15	4161	20,136	25,539
19903	35	♂	33,120	14,427	44	1797	9112	11,825
19905	30	♂	33,120	10,474	32	311	4409	12,231
19906	35	♀	33,120	4139	13	1422	3674	4729
19907	40	♂	33,120	1512	5	1276	8796	12,406
19908	40	♀	33,120	7549	23	1235	4491	6333
19909	35	♂	33,120	6204	19	417	3104	4916
19947	30	♀	33,120	5375	16	1552	6257	8292

*Note:* The age and sex of each individual were determined in the field during the process of fitting collars to the animals. The total number of fixes expected was determined based on the study period, the time collars stopped sending data due to functionality problems, and the time the animal died. The percentage of fixes received was calculated based on the proportion of expected fixes to actual fixes received. Symbol ♀ indicates female sex elephant and ♂ indicates male sex elephant.

**TABLE A2:** Coefficients of average movement speeds (km/h) by time of day, season, and protection level in isolation and combination that were derived from the linear mixed model. The study period spans 46 months (~4 years), comprising 1380 days, and encompasses four distinct dry and wet seasons.

	Estimate	Standard error	*T* value	*p*
Intercept	0.410	0.010	80.250	< 0.001
Low	0.070	0.010	9.990	< 0.001
Medium	0.050	0.010	5.340	< 0.001
Wet	0.110	0.010	15.910	< 0.001
Night	−0.050	0.010	−8.060	< 0.001
Low:wet	−0.060	0.010	−5.570	< 0.001
Medium:wet	−0.080	0.010	−6.320	< 0.001
Low:night	0.290	0.010	33.190	< 0.001
Medium:night	0.030	0.010	2.550	0.010
Wet:night	−0.120	0.010	−13.490	< 0.001
Low:wet:night	−0.140	0.010	−11.220	< 0.001
Medium:wet:night	−0.020	0.020	−1.000	0.320
