# Supplementary material for: Elephant Movement Data Provides Insights Into Conservation Challenges and Successes in the Ruaha–Rungwa Landscape
Source: Ecol Evol. 2025 Sep 30;15(10):e72197. doi: 10.1002/ece3.72197 (PMC12484299; doi:10.1002/ece3.72197)
Supplement: Supplementary file 1 — Data S1: ece372197‐sup‐0001‐DataS1.docx. [file ECE3-15-e72197-s001.docx]

**Supplemental 1**. Table showing attributes of the collared elephant individuals in the Ruaha-Rungwa ecosystem

| **ID** | **Collaring Date** | **Estimated Age** | **Sex** | **Tusk length** |
| --- | --- | --- | --- | --- |
| 19893 | 04-Nov-15 | 30 | Female | No tusks |
| 19888 | 04-Nov-15 | 35 | Female | No tusks |
| 19881 | 05-Nov-15 | 40 | Male | No tusks |
| 19892 | 05-Nov-15 | 30 | Female | No tusks |
| 19894 | 05-Nov-15 | 35 | Female | No tusks |
| 19885 | 05-Nov-15 | 40 | Male | 35 cm |
| 19906 | 06-Nov-15 | 35 | Female | No tusks |
| 19947 | 06-Nov-15 | 30 | Female | No tusks |
| 19880 | 07-Nov-15 | 25 | Female | 30 cm |
| 19907 | 07-Nov-15 | 40 | Male | 50 cm |
| 19895 | 07-Nov-15 | 35 | Female | 34 cm |
| 19886 | 07-Nov-15 | 40 | Male | 82 cm |
| 19897 | 08-Nov-15 | 30 | Female | No tusks |
| 19884 | 08-Nov-15 | 35 | Female | No tusks |
| 19908 | 09-Nov-15 | 40 | Female | No tusks |
| 19882 | 09-Nov-15 | 35 | Female | No tusks |
| 19890 | 10-Nov-15 | 30 | Female | No tusks |
| 19909 | 10-Nov-15 | 35 | Male | 46 cm |
| 19905 | 10-Nov-15 | 30 | Male | 45 cm |
| 19902 | 11-Nov-15 | 30 | Female | No tusks |
| 19889 | 11-Nov-15 | 30 | Female | No tusks |
| 19891 | 12-Nov-15 | 30 | Female | 44 cm |
| 19899 | 12-Nov-15 | 40 | Female | No tusks |
| 19883 | 12-Nov-15 | 30 | Female | 34 cm |
| 19900 | 13-Nov-15 | 40 | Male | 50 cm |
| 19901 | 14-Nov-15 | 35 | Female | No tusks |
| 19896 | 14-Nov-15 | 25 | Female | No tusks |
| 19903 | 14-Nov-15 | 35 | Male | No tusks |
| 19887 | 14-Nov-15 | 40 | Female | No tusks |
| 19904 | 15-Nov-15 | 20 | Male | No tusks |
